# Experimental evidence for anisotropic double exchange interaction driven anisotropic transport in manganite heterostructures

**DOI:** 10.1038/s41598-017-02813-x

**Published:** 2017-06-01

**Authors:** Z. L. Liao, G. Koster, M. Huijben, G. Rijnders

**Affiliations:** MESA+ Institute for Nanotechnology, University of Twente, P.O. BOX 217, 7500 AE Enschede, The Netherlands

## Abstract

An anisotropic double exchange interaction driven giant transport anisotropy is demonstrated in a canonic double exchange system of La_2/3_Sr_1/3_MnO_3_ ultrathin films epitaxially grown on NdGaO_3_ (110) substrates. The oxygen octahedral coupling at the La_2/3_Sr_1/3_MnO_3_/NdGaO_3_ interface induces a planar anisotropic Mn-O-Mn bond bending, which causes a significant anisotropic overlap of neighboring Mn orbitals. Due to the anisotropic double exchange interaction, it is found that the conductivity of the La_2/3_Sr_1/3_MnO_3_ film is enhanced when current is applied along the in-plane short crystalline axis. However, the anisotropic behavior is absent in the high temperature paramagnetic phase. Our results demonstrate anisotropic transport in the clean limit where phase separation is absent and the role of anisotropic phase percolation can be excluded.

## Introduction

Among the correlated electron materials much attention has been given to the manganites due to the exhibited spin polarization and colossal magnetoresistance. Although the richness and complexity of manganites are widely believed to originate from subtle interplay between spin, charge, orbital and lattice^1^, the true nature of their electronic behavior is still under debate and tremendous efforts are being paid to solve this challenging problem^2–4^. The double exchange (DE) mediated electron hopping^5, 6^, polaron dressed by lattice distortion^7, 8^ and phase separation^3, 9^ are the main proposed models to explain the observed metal-to-insulator transition and colossal magnetoresistance in manganites. Among these, the DE model lies at the origin of the exhibited ferromagnetic and metallic behavior^2–6^.

Furthermore, the transfer integral which depends on the overlap between oxygen 2p and Mn 3d orbitals determines the energy scale of the DE^5, 6^. In a manganite ruled by the DE electron conductivity will be strongly influenced by the transfer integrals, σ = (*xϵe* ^*2*^ */ahkT*), where, ϵ is the exchange energy, *x* is the doping level and *a* is Mn-Mn distance^5^. Since *ϵ* ≈ *kT*_*C*_ and *T*_C_ is the Curie temperature, the conductivity will be proportional to the *T*_C_ and σ = (*xe* ^*2*^ */ah*)(*T*_*c*_ */T*), which agrees well with the experimental observations^10, 11^. The DE model hypothesizes that an anisotropic DE will lead to anisotropic resistivities (AR) as shown by theoretical simulations^12^. Several experimental studies have demonstrated such anisotropic behavior using elastic anisotropic strain effect in La_5/8−*x*_Pr_*x*_Ca_3/8_MnO_3_ (LPCMO)^13^ and Pr_0.65_(Ca_0.7_Sr_0.3_)_0.35_MnO_3_ (PCSMO)^14^ thin films. Nevertheless, the giant anisotropic transport shown in LPCMO and LCSMO films, exhibiting metallic and insulating phase separation, are caused by structural distortion driven anisotropic phase nucleation and percolation^13^. In contrast to LPCMO and PCSMO which have sub-micrometer phase separation, La_2/3_Sr_1/3_MnO_3_ (LSMO) is recognized as a canonical DE system and is an ideal material for investigating anisotropic DE induced AR in the clean limit without the occurrence of the phase separation. Recently, AR was shown for a LSMO film grown on a DyScO_3_ (110) substrate due to a charge transfer from oxygen p_x_ to p_y_ orbitals induced by highly anisotropic tensile strain^15^. However, the large tensile strain in such LSMO films drives them into an insulating A-type antiferromagnetic ground state^15^. In this work, we present experimental evidence for anisotropic DE driven highly AR in ferromagnetic and metallic LSMO ultrathin films by employing the interfacial oxygen octahedral coupling (OOC) effect^16, 17^. In contrast to anisotropic strain^15^, which is either so large as to destroy the ferromagnetic ground state or too small to induce anisotropic transport, the OOC is found to result in a significant anisotropic crystalline structure and therefore strong anisotropic overlap of Mn orbitals, leading to giant anisotropic transport.

## Results

**Figure 1:**
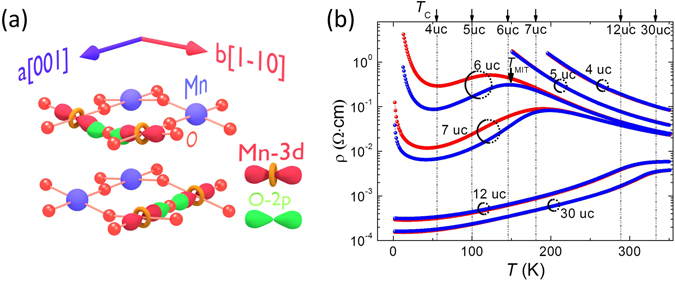
(**a**) Schematic view of anisotropic overlap of Mn-3d and O-2p orbitals along two in-plane orthogonal direction *a* (=[001]) (bottom panel) and *b* (=[1–10]) (top panel). (**b**) Temperature dependent resistivities with current along *a* (blue curves) and *b*-axes (red curves) for different thickness of LSMO films. The arrows indicate the Curie temperature of the films.

The LSMO films were grown on NdGaO_3_ (NGO) (110) substrates by pulsed laser deposition^17^. The orthorhombic (110) substrate is equal to pseudocubic (001)_pc_. All films are fully in-plane strained to the NGO substrates^17^ and thus the lattice constant of LSMO films along [001] and [﻿1–1﻿0] directions are 3.854 Å and 3.863 Å respectively^18^, leading to in-plane anisotropic strain of 0.2%. Our previous reports demonstrated a sharp interface between LSMO/NGO and uniform valence profile across the LSMO films^17, 19^. Due to the interfacial OOC effect, a structure with Glazer notation of b^+^a^−^c^−^ appears in LSMO in the region near to the interface in contrast to the strain driven a^+^b^−^c^−^ further away from the interface^17^. Therefore, the bond angle (θ) along the *a*-axis (=[001]) θ(*a*) is larger than along the *b* ^−^axis (=[1–10]) θ(*b*) in ultrathin films as schematically shown in Fig. 1a. For films with thicknesses above 8 unit cells (uc) the bond angle along the *a*-axis is smaller than along the *b*-axis, θ(*a*) < θ(*b*). The lateral anisotropic bond angle is expected to give rise to anisotropic Mn 3d and O 2p orbital hybridization as sketched in Fig. 1a.

Directly coupled to the strongly anisotropic crystal structure, giant transport anisotropy is observed in ultrathin LSMO films. The temperature dependent resistivities along the two orthogonal in-plane directions *a* and *b* measured by four-probe method are shown in Fig. 1b. A giant AR appears in 6 and 7 uc films at low temperatures, but is absent in thicker films (12 and 30 uc).

**Figure 2:**
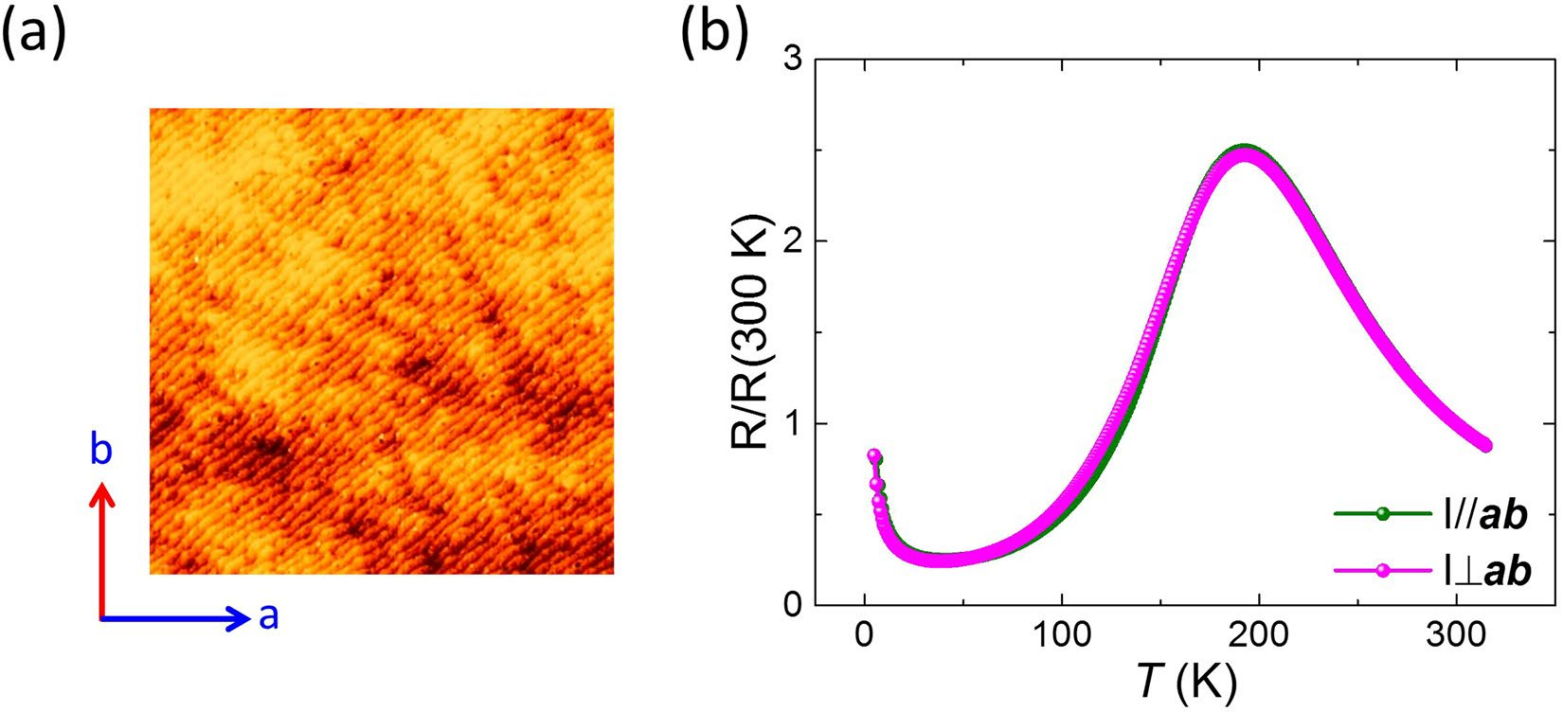
(**a**) Surface morphology of the 7 uc LSMO by AFM. (**b**) Resistivities of 7 uc LSMO film with current parallel or perpendicular diagonal direction *ab* (I//*ab* vs. I⊥*ab*).

Note that the AR measured by four-probe method is consistent with our previous report where the resistivity was obtained in a van der Pauw geometry^17^, though quantitatively different due to the fact that the van der Pauw method amplifies the anisotropic transport^20^. In addition, the absence of anisotropy for thicker LSMO films matches a previous study^15^, suggesting that the anisotropic elastic strain enforced by NGO substrate is too weak to induce AR, while the anisotropic OOC can be much more significant than strain in ultrathin films. Finally, the observed transport anisotropy should be related to the orientation of the crystal structure and is not dependent on the orientation of the step and terrace structure of the initial substrate surface. As shown in Fig. 2a, the orientation of the step edges of a 7 uc LSMO film is found to be along the diagonal direction (***ab***) of the square sample, while the resistivities and metal to insulator transition temperatures (*T*_MIT_) with currents parallel (I_//_) and perpendicular (I_⊥_) to the ***ab*** direction (see Fig. 2b). If the step edges should play a role in the observed giant anisotropic transport as observed for 2 dimensional electron gas at LaAlO_3_/SrTiO_3_ interface^21^, then one would expect a very large difference between R_//_ and R_⊥_, since the current for R_//_ and R_⊥_ are parallel and perpendicular to step edge, respectively.

The observed enhanced conductivity along the short *a*-axis is opposite to the expected long axis according to an anisotropic strain effect^13–15^, but is consistent with the interfacially driven anisotropic b^+^a^−^c^−^ structure^17^. Since with increasing film thickness the OOC effect will subside and the small anisotropic strain (0.2%) will gradually dominate, the AR will become weaker for thicker LSMO films. On the other hand, one would expect a more significant anisotropic transport in the thinnest films. However, the 4 uc LSMO is highly insulating and below 200 K the resistivity is out of the measurement range, as shown in Fig. 1b. The anisotropic transport in 6 uc LSMO films also disappears in the high temperature paramagnetic (PM) phase, in good agreement with the high temperature isotropic transport in the insulating 4 uc LSMO films.

The temperature dependence of the anisotropic transport behavior was studied by comparing the critical temperature (*T*_A_) at which the anisotropy starts to develop with the Curie temperature (*T*_C_). No sharp transition from isotropic transport to anisotropic transport occurs, therefore, the *T*_A_ is estimated to be the *T*_MIT_ for resistivity with the current applied along *a*-axis (see Fig. 1b). The *T*_C_ is determined from the temperature dependent saturated magnetization^17, 19^ and is indicated in Fig. 1b as well. Interestingly, the *T*_A_ is nearly equal to the *T*_C_ indicating a direct relation between the anisotropic electronic structure and the ferromagnetic metallic (FMM) state. These results provide experimental evidence of anisotropic double exchange interaction induced transport anisotropy as suggested by Dong *et al*.^12^.

**Figure 3:**
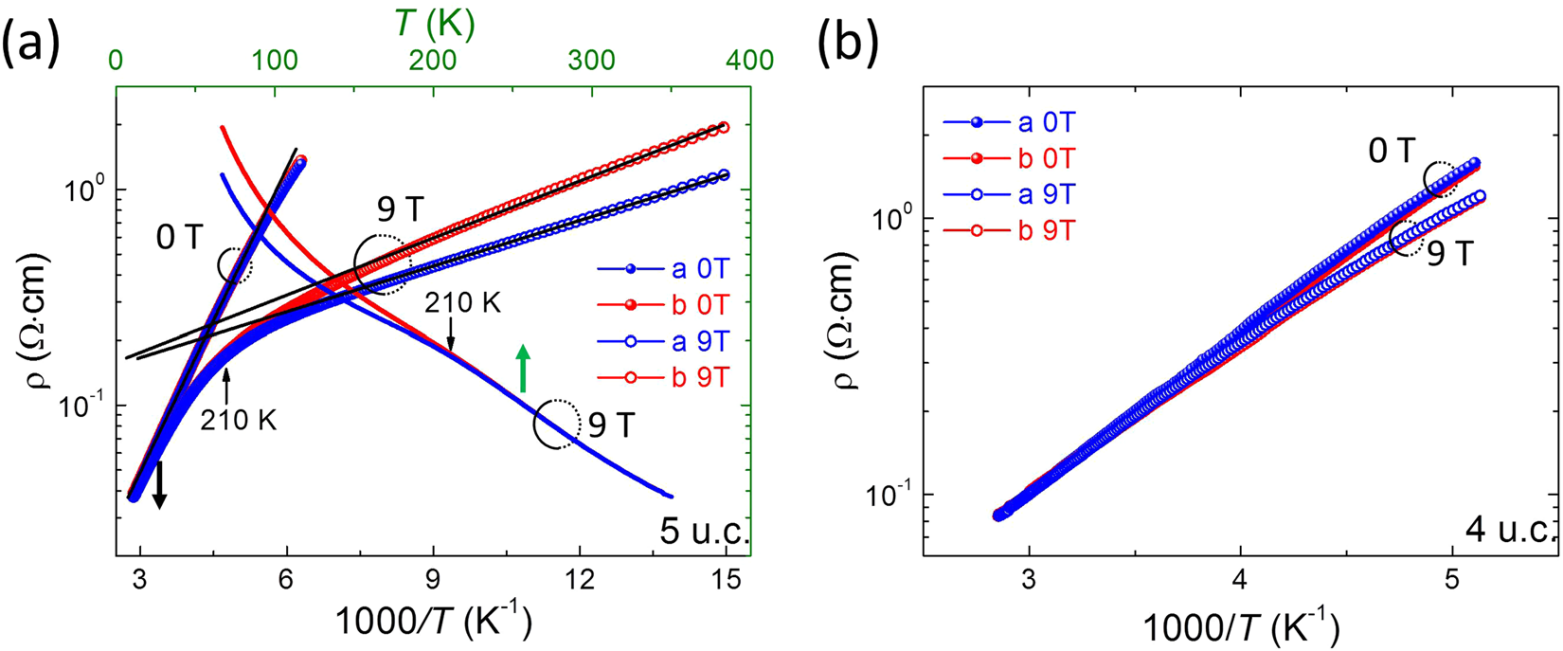
Log plot of resistivity along *a* and *b*-axes as a function of 1000/*T* and/or *T* for (**a**) 5 uc LSMO films and (**b**) 4 uc LSMO film with and without 9T magnetic field. The magnetic field was along out of plane direction.

To confirm that it is indeed the double exchange interaction playing a central role in the induction of the anisotropic transport, one expects anisotropic transport by tuning the paramagnetic state in LSMO films into the ferromagnetic metallic (FMM) state. To test this, we measured the magnetic field dependent transport anisotropy. As shown in Fig. 3a, a 9 T magnetic field drives the 5 uc LSMO film to be more conductive and *T*_*A*_ can be determined at about 210 K, below which the anisotropic transport starts to develop. The conductivity with current along *a*-axis was higher compared to the current measured along *b*-axis, exhibiting the same direction for higher conductivity seen in the 6 and 7 uc films. The magnetic field in the 5 uc LSMO film resembles the expected anisotropic transport induced by OOC.

The magnetic measurements also allow us to further illustrated the relation between aforementioned *T*_A_ and the electronic phase transition temperature by an Arrhenius-type plot of the resistivity as shown in Fig. 3a. At zero field, the Log(*ρ*) vs. 1/*T* curve is almost linear in the measureable temperature range, but with a 9 T external magnetic field, there is a transition at around 210 K where the thermal activation energy for electronic transport with I//*a*-axis (I//*b*-axis) is reduced from 88 meV (88 meV) to 14 meV (18 meV). In contrast, the 4 uc is still highly insulating and exhibits no transition under 9 T field (see Fig. 3b). As a result, no evident AR is observed in 4 uc LSMO even under high magnetic fields.

**Figure 4:**
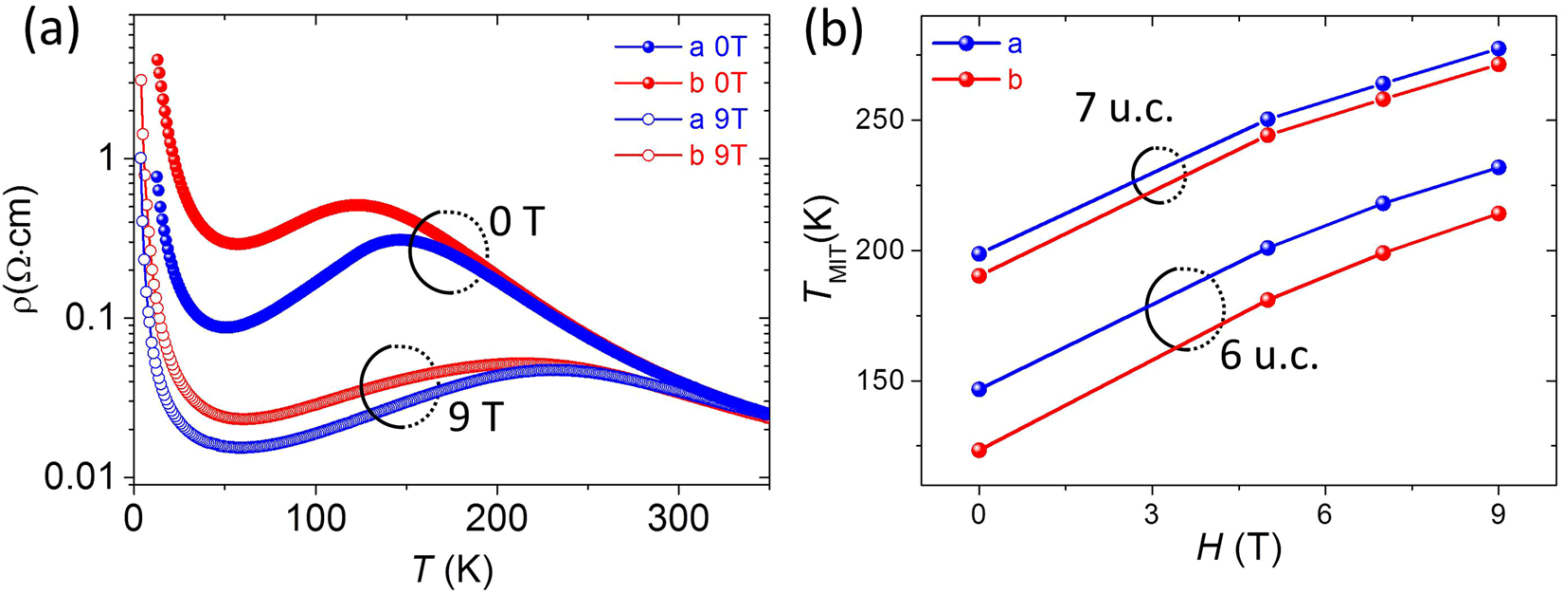
(**a**) Resistivity of 6 uc LSMO along *a* and *b*-axes as a function of temperature with and without 9 T magnetic field. (**b**) Magnetic field dependent *T*_MIT_ for 6 and 7 uc LSMO films. The magnetic field was along out of plane direction.

Figure 4a shows the magnetic field dependent AR in a 6 uc LSMO film. It is found that the *T*_MIT_ is shifted to a higher temperature after applying a 9 T magnetic field inducing an enhanced conductivity. Figure 4b shows the field dependent *T*_MIT_ of both 6 and 7 uc LSMO films with the current along the *a* and *b*-axes. For both directions *T*_MIT_ monotonically increases with magnetic field, while their difference Δ*T*_MIT_ = *T*_MIT_[*a*] − *T*_MIT_[*b*] remains almost constant.

The observed behavior in magnetic field is quite different as compared to studies on LPCMO whose Δ*T*_MIT_ converges zero with increasing magnetic field^13^, strongly excluding the role of elastically driven anisotropic phase percolation in our films. There is also no thermal hysteresis observed in the resistivity curves during the cooling-warming cycle, suggesting the absence of any sub-micrometer phase separation. The fact that the AR always starts to develop at the same temperature where the film enters the metallic phase further suggests the central role of anisotropic DE in transport.

Finally, the 6 and 7 uc LSMO films became insulating below ~50 K due to Anderson localization effect^22^, where there is still a finite density of states at the Fermi level and the conductivity arises from double exchange mediated electron hopping. Since the DE plays a central role in inducing conductivity and the room temperature OOC effect is expected constant while cooling down^23^, one is still able to observe the giant AR even for the low temperature ferromagnetic insulating phase. This is in strong contrast to the high temperature PM phase where conduction arises from thermal activation. The low temperature giant AR is also different from the AR observed in phase separated LPCMO and PCSMO films^13, 14^ in which the resistivity gradually becomes isotropic with decreasing temperature. This suggests that the AR in our ultrathin LSMO films grown on NGO (110) substrates is a clean DE driven transport anisotropy.

In conclusion, giant transport anisotropy has been induced in LSMO films by using interfacial structure engineering through the OOC effect. The OOC driven anisotropic structure leads to an anisotropic hybridization of the oxygen 2p and Mn 3d orbitals. Transport measurements show that the anisotropy develops in the FMM phase and disappears in the high temperature PM phase. Together with field dependent anisotropic behavior, our results demonstrate that the anisotropic exchange integral plays a pivotal role in anisotropic transport when double exchange dominates the conduction. Our results also suggest the vital role of Mn-O-Mn bond angle in affecting the double exchange transfer and electron conduction and provide an example to create novel properties by interfacial structure engineering.

## Methods

The LSMO films were grown on NdGaO_3_ (NGO) (110) substrates by pulsed laser deposition, for details see reference^17^. The temperature dependent resistivities and magneto resistance along the two orthogonal in-plane directions *a* and *b* were measured by four-probe method in a Quantum Design physics properties measurement system (QD-PPMS).
